# ROBOT-ASSISTED THERAPY FOLLOWING STROKE: WHAT EFFECTS ON QUALITY OF LIFE, COGNITIVE AND PSYCHOSOCIAL OUTCOMES? A SYSTEMATIC REVIEW

**DOI:** 10.2340/jrm.v58.44943

**Published:** 2026-02-11

**Authors:** Francesco ZANATTA, Alessandra GORINI, Luca FIORENTINO, Silvia TRAVERSONI, Cira FUNDARÒ, Marco D’ADDARIO, Patrizia STECA

**Affiliations:** 1 Department of Psychology, University of Milano-Bicocca, Milan; 2 Department of Clinical Sciences and Community Health, Department of Excellence 2023-2027, University of Milan, Milan; 3 PsyCaRe Lab, Istituti Clinici Scientifici Maugeri IRCCS, Milan; 4 Neurophysiopathology Unit, Istituti Clinici Scientifici Maugeri IRCCS, Montescano Institute, Montescano, Italy

**Keywords:** stroke, rehabilitation, robot-assisted therapy, systematic review, cognition, quality of life

## Abstract

**Objective:**

Robot-assisted therapy (RAT) has shown promise in post-stroke motor recovery. However, its effects on non-motor outcomes remain unclear. This systematic review evaluated RAT impact on post-stroke quality of life (QoL), cognition, and psychosocial functioning.

**Methods:**

Following PRISMA guidelines, electronic searches were performed from Web of Science, PubMed, Cochrane Library, CINAHL, Embase, and PsycINFO. Risk of bias was assessed using NIH Quality Assessment Tools. Data on study design, participants, intervention characteristics, outcomes, and results were extracted and synthetized descriptively.

**Results:**

A total of 90 studies met the inclusion criteria. Considerable heterogeneity was found in participants’ characteristics, intervention duration (2–52 weeks), and dosage (20–240 min/session). Most studies reported significant RAT effects on QoL (emotional, physical, cognitive, social subdomains), cognition (attention, executive functions, memory, language, visuo-spatial abilities, intelligence), and psychosocial outcomes (anxiety, depression, self-efficacy, fear of falling, motivation, coping). Some studies also showed greater improvements compared with conventional training controls. Longitudinal effects were generally absent, except for QoL variations observed up to 12 months. Cognitive and psychological factors were also identified as moderators/predictors of RAT response.

**Conclusion:**

Despite variability across studies, findings suggest RAT may have a broad impact beyond motor recovery. Future large-scale, standardized, longitudinal trials are recommended to confirm these results.

## LAY ABSTRACT

After a stroke, many people struggle not only with movement but also with thinking, emotions, and social life. Robot-assisted therapy is a new rehabilitation approach that helps patients perform repeated and precise movements with robotic support. We reviewed the studies of the last 2 decades to understand whether this therapy also improves aspects of life beyond movement. The results show that robot-assisted therapy can have positive effects on quality of life, mood, memory, attention, and well-being. Some studies even found better outcomes than with traditional rehabilitation. However, the improvements did not always last over time, and the studies differed in duration and intensity. Overall, our findings suggest that robot-assisted therapy could support more complete recovery after stroke, addressing both body and mind. Further research is still needed to confirm its long-term benefits for everyday clinical practice.

Over the past decades, population ageing has been increasing worldwide, contributing to a progressive rise in the incidence of various complex and chronic diseases, including stroke. According to the most recent global estimates, stroke accounted for approximately 93.8 million (89–99.3) cases and 7.3 million (6.6–7.8) deaths. Its impact, however, extends far beyond mortality and has presented significant challenges related to disability and quality of life (QoL) by accounting for a total of 160 million (148–172) Disability-Adjusted Life Years (DALYs) worldwide and an increase of 32.2% (21.7–42.6) over the last 20 years (1). Such global trends, along with the projected increase in stroke survivors, not only underscore the growing demand for long-term care and efficient rehabilitation services, but also focus attention on the necessity of addressing patients’ global functioning, as conceptualized within a biopsychosocial framework encompassing body structures and functions, activities, and participation in everyday life (2). Accordingly, given the multifaceted consequences of stroke, comprehensive, impactful, and sustained strategies are increasingly required.

Over the last years, the field of neurorehabilitation has increasingly embraced technology innovation to improve recovery outcomes (3). A prominent approach is robot-assisted therapy (RAT), which has gained considerable attention for its ability to enable highly tailored rehabilitation protocols along with repetitive, intensive, and task-oriented exercises that align with the principles of motor relearning and neuroplasticity (4, 5). RAT devices, including exoskeletons, end-effector systems, and soft-robots have so far provided customizable and controlled training paradigms that have efficiently supported the recovery of both upper and lower limb functions (6). Exoskeletons are wearable robotic systems composed of mechanical and electronic components that fully or partially align with a patient’s limb segment, guiding and assisting movement according to predefined kinematic or dynamic trajectories. End-effector devices, in contrast, interface only with the distal part of the limb, allowing movement to emerge more freely through the activation of a patient’s residual motor control, with fewer biomechanical constraints and a greater number of degrees of freedom. Soft-robots represent a more recent category of lightweight and flexible wearable devices, originally developed to support activities of daily living (ADLs) but increasingly applied in rehabilitation due to their adaptability and comfort. Compared with conventional motor training, these robotic approaches allow greater training intensity, precise control of movement parameters, and enriched task-oriented feedback. More recently, a growing body of research suggested that RAT can extend its potential beyond motor recovery, too. The repetitive and goal-directed nature of RAT, often combined with augmented visual or sensory feedback, may stimulate cognitive domains such as attention, executive functions, and learning processes through shared neural networks underlying motor and cognitive control. Moreover, increased patient engagement, motivation, and perceived self-efficacy during enhanced training may positively influence psychological outcomes. These advancements are particularly evident in the context of stroke rehabilitation, where RAT has shown promise to address a wider spectrum of patient symptoms, including cognitive deficits, functional impairment, and reduced psychosocial well-being (7–9).

Patients recovering from stroke often exhibit heterogeneous functional profiles, with cognitive impairments across various domains significantly impacting overall recovery. Commonly affected areas include attention, executive function, memory, language and visuo-spatial abilities, although the evidence on the domain-specific cognitive effects of rehabilitation remains limited (10, 11). Additionally, mental health issues are prevalent, encompassing conditions such as anxiety, depression, and inertia (12, 13). Taken together, cognitive and psychological symptoms can profoundly influence rehabilitation outcomes, often reducing treatment adherence and limiting patient activity, participation, and functional improvements (14). In response to these complexities, rehabilitation has increasingly embraced a broader perspective, incorporating non-motor domains into recovery processes. Studies have highlighted that RAT, particularly when integrated with supplementary systems (e.g., virtual reality [VR], wearable devices, functional electrical stimulation [FES], Brain–Computer Interface [BCI]), can enhance conventional training by offering a combined approach to post-stroke rehabilitation (15–17).

Nevertheless, despite the promising advancements in RAT over the last 2 decades, its integration into routine clinical practice remains limited due to a persistent gap between technological innovation and its standardized adoption within rehabilitation settings (3, 18), representing a key conceptual challenge in the current landscape of post-stroke rehabilitation. Moreover, while RAT has widely demonstrated its efficacy in motor recovery, recent literature still highlights the need for a deeper study of its widespread impact on non-motor dimensions (19), as evidence on QoL, cognitive, and psychosocial outcomes remains fragmented and often derives from secondary or exploratory analyses rather than being the primary focus of intervention trials. In addition, previous studies report substantial heterogeneity in outcome measures, intervention characteristics, and follow-up assessment, limiting the comparability of findings and the generalizability of conclusions across non-motor domains (9, 14, 17, 19). These gaps underscore the need for a systematic evaluation and synthesis of the broader effects of RAT to align its application with emerging patient-centred and technology-enabled healthcare models.

Following this line, the present work aimed to systematically review the available literature investigating the effects of RAT on post-stroke non-motor outcomes, namely QoL, cognition, and psychosocial functioning. Specifically, intervention procedures and metrics adopted to estimate RAT effects were summarized to provide future studies and rehabilitation practice with clearer evidence. Also, both short- and long-term effects were analysed to shed light on RAT’s lasting benefits and potentially inform future practices in post-discharge programmes.

## Methods

Preliminary checks on registered or ongoing similar literature reviews and/or meta-analyses were carried out through the International Prospective Register of Systematic Review (PROSPERO) platform. The search provided no results and, thus, the systematic review protocol was registered (ref. CRD42024594665).

### Search strategy and studies selection

The Preferred Reporting Items for Systematic Reviews and Meta-Analyses (PRISMA) guidelines (20) were followed throughout the entire review process. Preliminary electronic searches were performed on the 28 June 2024 from Web of Science, PubMed, Cochrane Library, CINAHL, Embase, and PsycINFO by applying the following search query: (rehabilitation) AND (stroke) AND ((robot*) OR (exoskelet*) OR (end-effector)) AND ((quality of life) OR (cogn*) OR (psych*) OR (well-being)). The use of general search terms as “rehabilitation” or “psych*” was preferred to ensure wider retrieval and select eligible records via a manual check along screening procedures. Eligibility criteria were defined *a priori* according to the PICOS framework, as follows: (P) adult patients with ischaemic or haemorrhagic stroke; (I) RAT delivered to upper and/or lower limbs, including exoskeletons, end-effector systems, and/or soft-robotic devices, either alone or coupled with supplementary systems (e.g., VR, FES, BCI); (C) Usual care, conventional rehabilitation, other technology-assisted interventions, or no-treatment control conditions; (O) post-stroke non-motor outcomes, namely QoL, cognitive functioning, and psychosocial variables; (S) experimental and observational study designs, including randomized controlled trials (RCTs), non-RCTs, before–after studies, and cohort studies. Moreover, additional filters (e.g., English language, original peer-reviewed research, year of publication timespan) were applied across electronic databases to optimize and refine the identification of the studies. In addition, the reference lists of all included full-text articles were manually screened to identify further relevant studies potentially missed by electronic search. A reference management system (HubMeta) (21) was used to import, find, and remove duplicates, and screen the records identified iteratively.

### Risk of bias assessment

All records included were evaluated based on their study design using the National Institutes of Health (NIH) Quality Assessment Tools for Controlled Intervention Studies (14 items), for Before–After (Pre–Post) Studies with No Control Group (12 items), and for Observational Cohort and Cross-Sectional Studies (14 items) (22). These included questions assessing criteria that ranged from study enrolment and evaluation (e.g., randomization, allocation, eligibility criteria, blinding, outcome measures validity) to intervention and analysis procedures (e.g., dropout management, adherence to treatment, power calculation, statistical analyses appropriateness). For all checklists, each item was rated as “yes” (1 point), “no” (0 points) or “not applicable”, resulting in a total score that was classified in terms of methodological quality as “Poor” (< 50%), “Fair” (50–75%), or “Good” (> 75%). Details on each tool’s questions and scoring procedures are reported as Supplementary material. Three researchers (FZ, LF, ST) evaluated all studies working independently and any discrepancies were discussed until full consensus was reached. The studies with lower scores (i.e., higher risk of bias) were not excluded from final synthesis, but their methodological quality was considered in interpretation of the findings (23).

### Data extraction and synthesis

According to eligibility criteria, progressive exclusion of non-eligible records was performed through screening procedures starting from the titles and abstracts, and then checking for the full texts. The entire review process was completed by 3 authors (FZ, LF, ST) blinded to one another’s studies classification. Disagreements on records exclusion were discussed and solved through planned periodical meetings with the entire research group aimed at reaching a consensus. At the full-text screening stage, each article identified was read multiple times to ensure a full understanding of the study aim, design, outcomes investigated, and findings. A wide range of data was then extracted from the studies included. These were collected in a synoptic table (see Supplementary material), which in detail includes: author(s), year of publication, country where the study was conducted and related Human Development Index (HDI) and rank (24), study design (including if pilot, funded, multicentre, and/or longitudinal), patients’ characteristics (i.e., type of hospitalization, stroke aetiology and onset, sample size, mean age, sex distribution, and ethnicity), RAT characteristics and delivery modalities (i.e., robotic device typology and name, targeted extremities, use of any supplementary device, overall trial duration, total number of RAT session and duration, and comparator(s)), study outcomes along with the metrics adopted (i.e., both motor and non-motor domains), and main results found. Descriptive statistics were computed on the main characteristics of the studies (i.e., means, standard deviations, percentages), while the results were meta-synthetized through a narrative approach. No statistical software or tool was employed for data analysis and synthesis.

## Results

### Studies selection

**Fig. 1:**
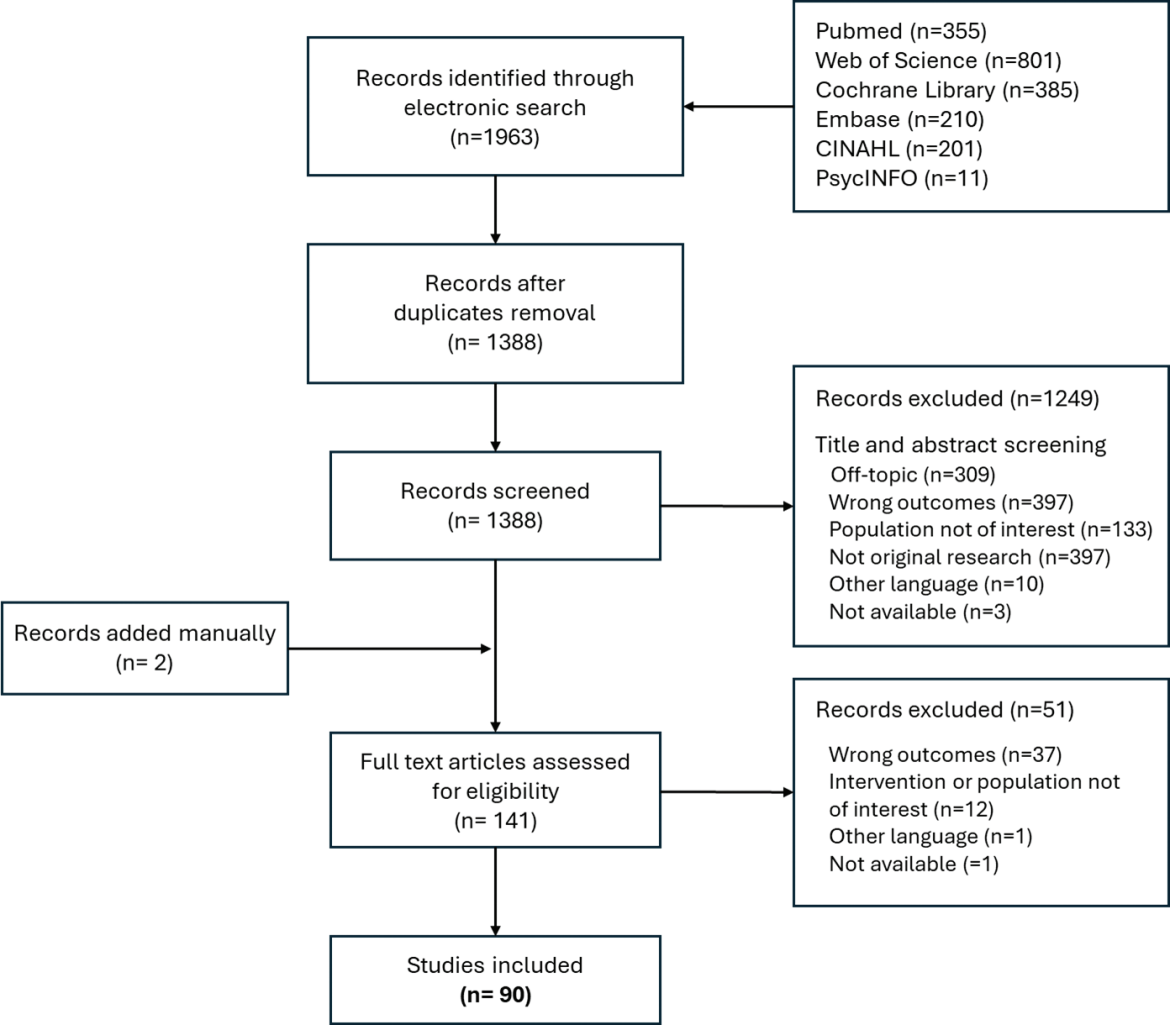
PRISMA flowchart.

At initial electronic search, a total of 1,963 records were retrieved. After duplicates removal, 1,388 records were screened by title and abstract. Two additional records were retrieved from abstract screening and added manually, for a total of 141 remaining articles that were screened by full text. Of these, 90 met all eligibility criteria and underwent data extraction. The review process and studies selection along with the reasons for exclusion at each screening stage are showed in Fig. 1. Most of the records excluded were labelled as off-topic (*n* = 309), included no non-motor outcomes (*n* = 434) or belonged to grey literature (*n* = 397). Others involved samples of paediatric patients or other clinical populations (*n* = 145), while fewer studies were excluded for language reasons (*n* = 11) or because the abstract or the full text was not available (*n* = 4).

### Risk of bias

The Controlled Intervention Studies checklist was used to evaluated both randomized controlled trials (RCTs) and non-randomized controlled studies (*n* = 61). The evaluation differed between the 2 study designs only for the items specifically referring to randomization procedures (items 1 to 3) and intention-to-treat analysis (item 14) criteria. These were labelled as “not applicable” for non-randomized controlled studies and were not counted for total scoring and subsequent quality rating. Overall, of the RCTs (*n* = 53), an equal distribution was observed between “Good” (*n* = 26, 49.1%) and “Fair” (*n* = 26, 49.1%) studies, with only 1 trial labelled as “Poor” (1.8%). Similarly, half of the non-randomized studies were rated as “Good”, the other half as “Fair” (*n* = 4, 50%). Regarding the Before–After (Pre–Post) studies with no control group (*n* = 10), most were classified as “Fair” (*n* = 8, 80%), whereas only 2 studies reached “Good” methodological quality (20%). Lastly, the Observational Cohort and Cross-Sectional Studies assessment tool was used to evaluate retrospective cohort studies (*n* = 19). The majority were rated as having “Fair” quality (*n* = 11, 57.9%), and fewer were classified as “Good” (*n* = 8, 42.1%).

Overall, satisfactory levels of methodological quality were observed across all the studies included. Only a small number (i.e., 11.1%) were rated as “Poor” or were at the lower marginal limits of “Fair” classification, thus potentially having a higher risk of bias. Across study designs, most recurrent methodological concerns regarded the absence of blinding of patients, treatment providers, and outcome assessors, the lack of *a priori* sample size or power calculations, the absence of stratified analysis exploring dose–response relationship, and the limited consideration of potential confounding factors. None of the studies included satisfied all evaluation criteria in any of the tools adopted. For each study, details on the evaluation are provided as Supplementary material.

### Characteristics of the studies included

All data extracted from the studies included are presented in a synoptic table (see Supplementary material) (25–114).

### Design and participants

The main characteristics and the design of the studies included are presented in **Tables I** **and** **II**. Almost half of the studies were published within the last 5 years (*n* = 43, 47.8%) and conducted in European countries (*n* = 44, 48.9%). In particular, most were from countries classified as having “very high” (86.7%) or “high” (12.2%) human development, according to the latest United Nations HDI rank. Regarding the design, the majority of conducted RCTs (58.9%), were single-centred (77.8%) and received funding (51.1%). Fewer studies were pilot (18.9%) and included follow-up evaluations (30.0%), which mainly lasted less than 6 months (59.3%, follow-up range: 2 weeks–72 months).

**Table I:** Main characteristics of the studies included (*n* = 90)

Year of publication	*n* (%)	Nation^a^	*n* (%)^b^	HDI rank^c^	*n* (%)
2021–2024	43 (47.8)	Europe	44 (48.9)	Very high	78 (86.7)
2016–2020	31 (34.4)	Asia	33 (36.7)	High	11 (12.2)
2000–2015	16 (17.8)	America	13 (14.4)	Medium	1 (1.1)
		Oceania	1 (1.1)	Low	–

a Europe (i.e., Lithuania, Italy, Austria, Germany, Sweden, France, Belgium, Czech Republic, Netherlands, United Kingdom, Switzerland, Spain, Romania), America (i.e., Canada, United States of America), Asia (i.e., China, South Korea, Japan, Turkey, Taiwan, Russia, India), Oceania (Australia).

b Non-cumulative percentage.

c The Human Development Index (HDI) is a composite score accounting for (*i*) life expectancy at birth, (*ii*) expected years of schooling and mean years of schooling, and (*iii*) Gross National Income per capita. Rank categorization is based on 2023–2024 United Nations Development Programme Report, https://hdr.undp.org/ (accessed November 1, 2024).

**Table II:** Main design characteristics of the studies included (*n* = 90)

Study design	*n* (%)	Follow-up	*n* (%)	Multicentre	*n* (%)	Pilot	*n* (%)	Funded	*n* (%)
RCT	53 (58.9)	Yes	27 (30.0)	Yes	20 (22.2)	Yes	17 (18.9)	Yes	46 (51.1)
Retrospective cohort	19 (21.1)	No	63 (70.0)	No	70 (77.8)	No	73 (81.1)	No	44 (48.9)
Non-RCT	8 (8.9)								
Pre–post clinical trial (no control group)	10 (11.1)								

Of the participants involved (**Table III**), most were inpatients (*n* = 62, 68.9%) and were diagnosed with ischaemic or haemorrhagic stroke (*n* = 72, 80.0%). Overall, a total of 6,521 patients were included. Some 26.7% of the studies (*n* = 24) included less than 30 participants (range: 7–770). Moreover, the majority involved samples aged on average under 65 years (*n* = 62, 68.9%) and were mostly composed of men (i.e., ≥ 60.0%; *n* = 66, 73.3%). Only 2 studies reported participants ethnicity.

**Table III:** Participants’ characteristics of the studies included (*n* = 90)

Hospitalization	*n* (%)	Stroke type	*n* (%)	Stroke onset	*n* (%)
Inpatients	62 (68.9)	Ischaemic and haemorrhagic	64 (71.1)	< 6 months	53 (58.9)
Outpatients	19 (21.1)	Ischaemic	8 (8.9)	> 6 months	29 (32.2)
Both	5 (5.6)	Chronic	10 (11.1)	Not defined	8 (8.9)
Not defined	4 (4.4)	Not defined	8 (8.9)		

### Intervention

The types of robotic devices used along with the main characteristics of the interventions are summarized in Table IV. Most of the included studies implemented exoskeletal devices (*n* = 48, 53.3%) followed by the use of end-effectors (*n* = 38, 42.2%) for targeting either upper (48.9%) or lower (50.0%) limb function recovery. The intervention period varied widely across the studies in terms of overall duration (range: 2–52 weeks), total number of sessions (range: 2–60), and session duration (20–240 min). Of the studies with control conditions, intervention effects were compared with patients assigned to usual care (*n* = 50, 63.3%) or other technology-assisted training or procedures (e.g., VR-based therapy, passive robotic intervention; different robotic device; *n* = 21, 36.7%). Of the total, 11 studies (12.2%) implemented supplementary devices in combination with RAT as experimental condition (i.e., functional and neuromuscular electrical stimulation, brain–computer interface systems, non-immersive VR devices, cycle ergometer, bodyweight support system-integrated treadmill).

**Table IV:** Main characteristics of the robotic devices implemented and of the intervention of the studies included (*n* = 90)

Robot typology	*n* (%)	Targeted extremities	*n* (%)	Intervention	Mean ± SD (range)
Exoskeleton	48 (53.3)	Upper limbs	44 (48.9)	Overall duration (weeks)	6.1 ± 5.6 (2 – 52)
End-effector	38 (42.2)	Lower limbs	45 (50.0)	No. of sessions	21.6 ± 9.9 (2 – 60)
Both	2 (2.2)	Not defined	1 (1.1)	Session duration (min)	51.9 ± 32.8 (20 – 240)
Not defined	2 (2.2)				

### Outcomes

**Fig. 2:**
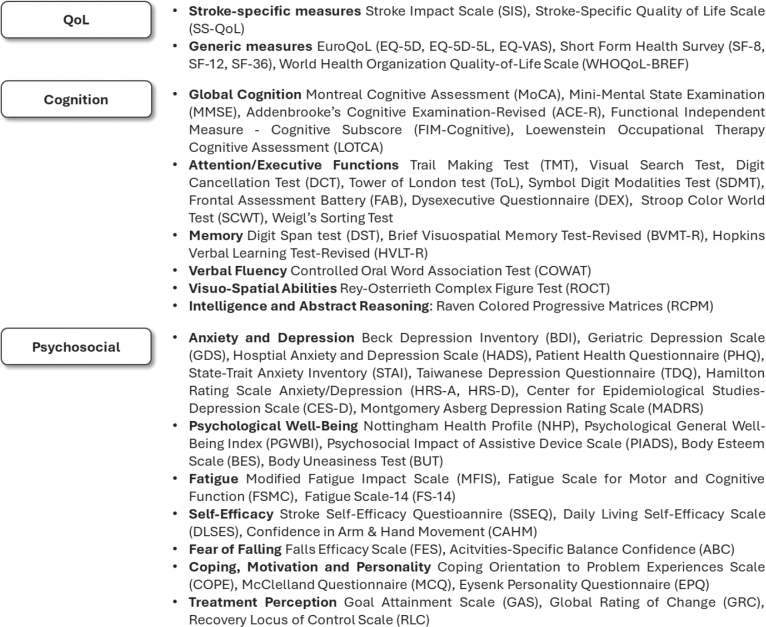
Summary of the non-motor outcome measures adopted in the studies included.

A summary of the non-motor outcomes and domains investigated is reported in Table V. These were evaluated through standardized measures intended to profile patients’ QoL, cognition, and psychosocial functioning, which were conceptualized in the present review as interrelated components of post-stroke global functioning, reflecting patients’ emotional, cognitive, behavioural, and social adaptation to daily activity and participation after stroke. Regarding QoL evaluation (*n* = 46, 51.1%), a wide range of sub-domains were investigated and, in the present review, classified as a measure of well-being, based on emotional (e.g., mood, role limitations due to emotional problems), physical (e.g., energy, fatigue), cognitive (e.g., memory, communication), or social (e.g., participation, work/productivity) factors, thus capturing patients’ perceived functioning across multiple life areas. As for cognitive functioning (*n* = 43, 47.8%), most of the studies conducted comprehensive evaluations on global cognition, while others assessed selected cognitive domains like executive functions, attention, memory, visuo-spatial abilities, and/or intelligence and reasoning, which underpin patients’ capacity to plan, adapt, and effectively engage in rehabilitation and daily activities. Of the studies including psychosocial variables (*n* = 37, 41.1%), most evaluated patients’ anxiety and/or depression symptoms, while the remaining specifically estimated intervention changes on perceived self-efficacy, fear of falling, psychological well-being, fatigue, coping strategies, and motivational factors, all of which reflect emotional regulation, personal resources, and psychological adjustment within the broader framework of global functioning. Outcome metrics for each study domain are summarized in Fig. 2.

**Table V:** Summary of the non-motor outcomes and domains investigated in the studies included (*n* = 90)

QoL^a^	*n* (%)^b^	Cognition	*n* (%)^b^	Psychosocial	*n* (%)^b^
Emotional well-being	32 (35.5)	Global functioning	32 (35.5)	Anxiety/Depression	29 (32.2)
Physical well-being	20 (22.2)	Attention	9 (10.0)	Self-efficacy	5 (5.5)
Cognitive well-being	30 (33.3)	Executive functions	14 (15.5)	Fear of falling	5 (5.5)
Social well-being	36 (40.0)	Memory	5 (5.5)	Psychological well-being	4 (4.4)
		Visuo-spatial abilities	3 (3.3)	Fatigue	3 (3.3)
		Intelligence	1 (1.1)	Treatment perception	3 (3.3)
				Coping	2 (2.2)
				Motivation	1 (1.1)
				Personality traits	1 (1.1)

a Emotional well-being (SF Health Survey – role limitation due to emotional problems, emotional well-being; SIS – emotion; SS-QoL – mood, personality; WHOQOL-BREF – psychological domain); Physical well-being (SS-QoL – energy, SF Health Survey – energy/fatigue; role limitation to physical problems; NHP – energy); Cognitive well-being (SS-QoL – language, thinking; SIS – memory, communication); Social well-being (SS-QoL – family roles, social roles, work/productivity; WHOQOL-BREF – social domain, environmental domain; SF Health Survey – social functioning; SIS – social participation).

Treatment perception includes outcomes related to intervention expectations, perceived effectiveness and recovery locus of control.

b Non-cumulative percentages.

### RAT effects on QoL

More than the half of the studies including QoL outcomes reported significant changes following RAT (*n* = 29, 63.0%), while fewer reported null effects (*n* = 17, 37.0%). Significant improvements were found in the subdomains of emotional (27, 33, 46, 58, 61, 70, 80–83, 85, 92, 102, 112), physical (27, 46, 54, 61, 70, 80, 82), cognitive (27, 33, 46, 58, 70, 80–83, 92, 112), and social (27, 33, 44, 46, 58, 61, 68, 70, 80–83, 85, 87, 92, 102, 103, 105, 106, 112) well-being, with some studies also reporting wider changes than a control group exposed to usual care (27, 33, 44, 54, 70, 80, 82, 85) or alternative therapy (46, 58, 68, 82, 87). On average, the overall duration of RAT in the studies estimating significant effects was on average 8 weeks (range: 3–48 weeks) and mainly deployed exoskeletal devices. Moreover, most studies assessed patients who initiated the intervention within 6 months after stroke onset (68, 4%) and included follow-up evaluation with varying duration (range: 1 month–6 years from RAT completion). Only 8 studies found significant longitudinal effects (26, 33, 44, 58, 85, 88, 102, 103). Of these, follow-up duration was up to 12 months and reported on all well-being subdomains.

### RAT effects on cognition

Most of the studies investigating RAT’s impact on cognitive outcomes found significant improvements (*n* = 26, 76.5%). Among these, most observed significant post-intervention changes on global cognition (25, 39, 41, 45, 63, 65, 76, 78, 97, 101, 104, 110, 113, 114), followed by within-subject effects on executive functions (31, 37, 56, 62, 69, 76, 78, 101), attention (25, 31, 37, 56, 78), visuo-spatial abilities (25, 31, 101), memory (25, 31), and intelligence (36). Additionally, 10 studies showed that RAT was significantly superior in targeting these outcomes compared with standard care (36, 37, 41, 45, 65, 78, 101) or other training methods (i.e., VR-based training, alternative robotic devices, gait training with orthoses) (25, 78, 96, 114). Overall, RAT duration ranged from 2 to 8 weeks, primarily using end-effector or exoskeleton devices coupled to non-immersive VR exposure. More than the half of the studies (60.0%) included patients who had experienced stroke 6 months prior to the intervention. Only 2 studies (73, 90) conducted follow-up evaluations at 4 and 8 -months following RAT, respectively, and neither found significant long-term effects.

### RAT effects on psychosocial outcomes

Approximately half of the studies investigating RAT’s impact on psychosocial outcomes reported significant effects (*n* = 18, 52.9%), while the other half did not. At post-intervention, significant within-group improvements were observed in anxiety or depression symptoms (35, 42, 51, 67, 72, 76, 78, 79, 88, 93, 98, 100, 111), psychological well-being (35, 42, 48, 76), coping strategies (35, 42), self-efficacy (38, 50, 100), fear of falling (88), and perceived fatigue (37, 51). Fewer studies reported significant group interaction effects, suggesting RAT to be more effective than standard training controls (42, 50, 51, 79, 85, 88, 98, 111). RAT overall duration ranged from 2 to 8 weeks and mainly employed the use of exoskeletons without any supplementary devices. Most of the studies included patients affected by stroke within the last 6 months (56.3%). Only 2 of them (67, 85) conducted follow-up evaluations (up to 4 months), but no significant effects over time were reported on psychological outcomes.

### Non-motor correlates of RAT effectiveness

Some of the studies included in data synthesis investigated the role of selected non-motor variables as covariates or predictors of patient recovery. Among these, significant correlations were found between motor improvement and global cognition (74, 77, 101), executive functions (29, 55, 93), attention (29), and visuo-spatial abilities (93). Specifically, 2 studies showed that global cognition at baseline significantly predicted increased patient autonomy (71, 77), whereas others reported no significant impact over the trial period (32, 89, 97). Similarly, significant associations were estimated with self-efficacy (75, 100), locus of control (34), depression (100), and anxiety (34) symptoms, which significantly predicted better rehabilitation outcomes.

## DISCUSSION

The present review aimed to systematically summarize the studies that investigated the efficacy of RAT on non-motor outcomes in patients affected by stroke. Specifically, from each study included, the effects on QoL, cognition, and psychosocial outcomes were extracted and meta-synthesized, with the final purpose of providing an overview of the widespread impact of robotics in post-stroke rehabilitation programmes.

At screening process completion, a total of 90 studies were analysed. Of these, most were published over the past 10 years with a notable increase in the last 5, indicating rapid and growing attention on using RAT to target a wider range of post-stroke outcomes besides motor functioning. Such interest has spanned multiple countries worldwide, particularly those at higher HDI levels. This not only reflects limited use of robotics in less developed countries, but also underscores the need to promote more equitable access. Given the global rise in stroke cases projected over the coming decades (2, 115), fostering wider accessibility to robotic technologies could be crucial. In light of this, considering also the higher costs associated with technology implementation compared with traditional treatments (more than the half of the studies included were funded), future research on RAT should integrate cost-effectiveness data into their analyses, thereby enriching the literature with additional insights for improved economic considerations. Beyond this, wider accessibility to robotic technologies may also be particularly relevant from a rehabilitation perspective. RAT allows the delivery of training with precision and consistency that is harder to achieve with conventional therapy alone. Among the advantages are high intensity, repetition, and task-specificity along with objective control and enriched sensorimotor feedback. These features may be especially valuable for patients with severe impairments or limited voluntary movement, for whom standard treatment can be less effective or more difficult to individualize.

Regarding the methodological characteristics of the studies included, heterogeneous and mixed data were synthetized. Although most of the works adopted an RCT design, many others reported retrospective cohort observations, did not perform randomization, or tested the efficacy of RAT with no control groups. Moreover, most were single-centred, did not schedule follow-up evaluations, and involved patients affected by stroke of different aetiology and varying time of onset. Sample size also varied extensively. Although a considerable number of patients were included in this review, 1 study in 4 involved less than 30 participants, often leading to statistically underpowered evidence. Furthermore, most patients were men and aged under 65 years. Although such distribution aligns with the global trends observed over the past 2 decades (i.e., among people younger than 70 years, prevalence and incidence rates increased by 22% and 15% respectively; males experienced a greater burden of disease in terms of DALYs compared with females) (116), this configuration constrains the drawing of conclusions concerning RAT’s effectiveness across other demographic groups, such as older adults and females. Lastly, only 2 studies reported participants’ ethnicity, which results in additional lack of informative data for findings’ replicability and applicability, as suggested in a prior study (117). Taken together, despite the heterogeneity observed, the overall methodological quality of the studies included was judged as satisfactory. Based on the risk-of-bias evaluation undertaken, more rigorous randomization methods, allocation concealment, blinding procedures, and intention-to-treat analyses are encouraged for future RCTs, when applicable. Also, analysing individual-level data along with the adoption of interrupted time-series designs is recommended for future studies with no control group to further increase results confidence and analysis sensitivity. Furthermore, future observational cohort studies should more accurately identify and measure key potential confounding variables so as to better investigate their impact on the relationship between RAT exposure and rehabilitation outcomes over time.

Further heterogeneity was found regarding the intervention characteristics. Despite the studies included being equally distributed for the use of device typology (exoskeleton or end-effector) and for targeted extremities (upper or lower) and related functions, the duration of the rehabilitation programmes varied widely in terms of total number of sessions and each session’s duration. These data reflect the broader and ongoing debate regarding the definition of treatment dosage. While it is acknowledged that training intensity can significantly impact post-stroke neural reorganization and rehabilitation outcomes, the optimal dosage threshold has not yet been established (118, 119). So far, the use of robotic technology has been considered valuable to increase the amount of therapy, as it allows better research into treatment dosage through more precise and quantifiable control of therapy (17). In the present review, across the data extracted, no clear and repeated patterns were found, showing that longer or more intensive interventions were linked to increased effectiveness across outcome domains. However, future meta-analyses should further address this association to better inform the dose–response relationship and seek proper treatment intensity for patients, specifically in targeting non-motor outcomes. Moreover, very few studies among those included investigated the role of treatment intensity or timed initiation as covariates of non-motor change. Further work in this direction is warranted. Following this line, future research should also adopt more theory-driven and mechanistic approaches to better clarify the potential transfer effects of RAT on non-motor outcomes. Standardized protocols and systematic manipulation of specific treatment components (e.g., task demands, feedback modalities) are needed to provide deeper insights into how different robotic deployments may differentially affect the non-motor recovery process.

Multiple outcome domains and measures were retrieved and synthetized. Moreover, intervention effects were examined with reference to the trial design and participants’ characteristics. Notably, all studies adopted standardized metrics, which ensured increased validity of the data collected across the different interventions. Of those assessing changes on QoL, both generic and stroke-specific tools were used. Widespread effects were observed on various aspects of perceived well-being, including emotional, physical, cognitive, and social sub-domains. These mainly provided encouraging evidence on the immediate impact of RAT (resulting in some cases being superior to standard training), while only a smaller proportion of studies supported the long-term effects after the intervention (up to 12 months). These findings corroborate what emerged from a prior systematic review on QoL changes following RAT (9), which supported its integration into conventional treatments but reported limited longitudinal evidence especially among individuals with stroke, despite this being the most investigated neurological population. Accordingly, further follow-up research is encouraged to enrich the literature with longitudinal evidence and draw more robust conclusions concerning RAT’s effects on perceived well-being over time. Moreover, the high variability observed in participants’ stroke aetiology and time since first onset made it difficult to identify possible clinical determinants of RAT efficacy on QoL. Future studies involving larger and more clinically homogeneous samples are recommended. Contextually, performing more in-depth cluster analyses and targeting selected well-being sub-domains would also be informative.

Regarding the studies focusing on cognitive outcomes, batteries of standardized tests were used. Pre–post intervention evaluations included global cognition, attention, executive functions, memory, visuo-spatial abilities, and intelligence. Again, significant changes following RAT were observed across all cognitive domains, with some studies showing the integration of robotics to provide wider improvements compared with traditional treatments. This finding confirms the potential of robotic technology to promote neuroplasticity and, simultaneously, provide cognitive stimulation. It is widely acknowledged that motor recovery not only relates to the motor processes but also to the sensory and proprioceptive systems, which share mutual neural circuits and influence each other during training (120, 121). By increasing treatment intensity, repeatability, and optimization, RAT enables augmented motor inputs from peripheral joints, ultimately providing task-specific stimulation to the central nervous system and a concurrent impact on cognitive functioning (122, 123). Moreover, most of the included studies combined the use of robotics with VR devices, which further enhanced patients’ exposure to multimodal and multisensory stimuli. Accordingly, the interaction with visual feedback or virtual games coupled to robot-assisted reaching, grasping, or walking improved dual-task training, which in turn facilitated the activation of the neuroplastic processes involved in the recovery of both motor and cognitive functions. Accordingly, future trials are encouraged to leverage the combination of RAT and VR. Despite being promising, however, it must be noted that the results synthetized were extracted mainly from studies including clinically mixed and small samples and describing interventions of varying durations. Additionally, none of them reported significant effects over time. Future full-scale and standardized intervention studies including more extended follow-up evaluations are recommended to better clarify the longitudinal benefits of RAT on cognition.

Finally, of the psychosocial outcomes included, a wide range of domains were investigated, namely depression and anxiety levels, self-efficacy, fear of falling, psychological well-being and fatigue, and coping strategies. Similarly to previous outcomes, significant effects were found following RAT across almost all outcomes, with major evidence on depression and anxiety changes. Other work also focused on the moderating or predictive role of motivational factors, perceived locus of control, and personality traits to impact rehabilitation outcomes, but mixed or inconclusive results were found. Interestingly, most of the studies that reported a positive psychological impact also evidenced significant improvements in motor and functional status. While this finding suggests that RAT may be effective for non-motor outcomes, evidence on the relationship between motor and psychological change was limited. This makes it difficult to determine whether the psychological benefits were secondary to motor improvement or a direct result of RAT exposure. In general, the psychosocial benefits resulting from neurorehabilitation processes are increasingly recognized, especially when multidisciplinary approaches are adopted (124, 125). Additional studies investigating the integration of robotics into standard training along with multi-domain network analyses are recommended to better outline the widespread impact of RAT. Furthermore, even in studies investigating psychosocial outcomes, the interventions varied widely in total duration and dosage intensity, with a predominant use of exoskeletons. Moreover, most of the patients involved had stroke onset within 6 months and, at RAT completion, were evaluated only up to 4 months, with no significant longitudinal effects being reported. Future studies with standardized intervention parameters are suggested to identify optimal protocols for RAT additionally targeting psychosocial outcomes. Extending follow-up periods and exploring RAT’s impact in later stroke stages is also crucial to determine long-term effects and broader applicability.

### Limitations and directions for future research

Overall, the studies included in this systematic review allow for the description of informative and encouraging evidence on robotic devices’ application to post-stroke rehabilitation programmes, especially the impact on non-motor outcomes. However, some limitations should be acknowledged. First, despite the relatively large number of studies identified, no meta-analysis was conducted. This choice was driven by the substantial heterogeneity observed across studies in terms of design, participants’ profile, intervention features, and outcome domains and measures, which precluded a reliable quantitative synthesis. Such variability also made it difficult to draw robust conclusions and represents the main limitation of the present review. Outcome-specific in-depth analyses along with the implementation of more adaptive RAT trial protocols are highly recommended for future studies. In addition, in most of the included studies non-motor outcomes were not defined as primary endpoints but instead assessed as secondary or exploratory measures. This may have resulted in limited statistical power, thereby constraining the findings’ interpretability and generalizability. Future research should prioritize more rigorous methodological designs (e.g., improved randomization, blinding, and longitudinal follow-up), larger and more clinically homogeneous samples, and extend follow-up periods to clarify dose–response relationships, long-term effects, and applicability across different stroke stages. Also, theory-driven and mechanistic studies manipulating key intervention components are needed to elucidate the transfer of RAT onto non-motor outcomes (as primary endpoints). Integrating cost-effectiveness analyses and adopting a more integrative, multi-domain, and network-based approach will be essential to advance personalized, patient-centred rehabilitation pathways and inform future clinical implementation. Also, the adoption of longitudinal designs is warranted. Prospective data would better inform RAT effectiveness through multiple observations, ultimately highlighting outcomes’ long-term trajectories and the role of potential correlates over time. In conclusion, future research should also adopt more integrative approaches. Besides motor functions, the assessment of QoL, cognitive, and psychosocial outcomes within the same intervention protocol would allow for more comprehensive evaluations of the multi-domain impact of RAT. This method would better address post-stroke global functioning, identify potential recovery profiles, and ultimately promote the development and implementation of individualized, patient-centred rehabilitation pathways.

### Conclusions

RAT shows promising effects beyond post-stroke motor recovery, potentially improving non-motor outcomes such as QoL, cognition, and psychosocial functioning. However, substantial heterogeneity limited generalizable conclusions, calling for more rigorous, complex, and theory-driven studies with long-term follow-up to better clarify mechanisms, effectiveness, and clinical implementation.

## Supplementary Material


